# Interrelationships between early antenatal care, health facility delivery and early postnatal care among women in Uganda: a structural equation analysis

**DOI:** 10.1080/16549716.2020.1830463

**Published:** 2020-10-30

**Authors:** Ruth Atuhaire, Leonard K Atuhaire, Robert Wamala, Elizabeth Nansubuga

**Affiliations:** a Department of Management Science, Makerere University Business School, Kampala, Uganda; b Department of Planning and Applied Statistics, Makerere University School of Statistics and Planning, Kampala, Uganda

**Keywords:** Generalized structural equation model, maternal healthcare, timely, utilization, interconnectedness

## Abstract

**Background:**

Early medical checkups during and after delivery are key strategies to detect, prevent and treat maternal health concerns. Knowledge of interrelationships between early Antenatal Care (ANC), skilled delivery and early postnatal care (EPNC) is essential for focused and well-targeted interventions. This paper investigated the interconnectedness between maternal health services in Uganda.

**Objective:**

This study examines the predictors of interrelationships between early antenatal care, health facility delivery and early postnatal care.

**Methods:**

We used a sample of 10,152 women of reproductive ages (15–49), who delivered a child five years prior to the 2016 Uganda Demographic and Health Survey. A generalized Structural Equation Model and STATA 13.0 software were used.

**Results:**

Early ANC was a mediating factor for health facility delivery (aOR=1.04; 95% CI=1.01-1.14) and EPNC (aOR=1.1; 95% CI=1.05-1.26). Increased odds of early ANC utilization was directly associated with: Adult women aged 35-49 (aOR=1.18; 95% CI=1.10-1.35), having completed primary seven (aOR=1.68; 95% CI=1.56-1.84); distance to a health facility (aOR=1.35; 95% CI=1.23-1.73) and costs (aOR=1.85; 95% CI=1.31-2.12) not being a problem, available community workers (aOR=1.06; 95% CI=1.04-1.17), pregnancy complications (aOR=2.04; 95% CI=1.85-2.26) and desire for pregnancy (aOR=1.15; 95% CI=1.07-1.36). Through early ANC utilization, being married (aOR=1.16; (=1.04*1.10)), no distance issues ((aOR=1.40; (=1.04*1.35)) and complications (aOR=2.12; (=1.04*2.04)) indirectly influenced utilization of health facility delivery. Women aged 20-34 (aOR=1.01; (=0.92*1.1)), completing primary seven (aOR=1.85; (=1.69*1.1)) and no cost problems (aOR=2.04; (=1.85*1.1)) indirectly influenced EPNC.

**Conclusion:**

Early antenatal care was a mediating factor for health facility delivery and EPNC; and hence, there is need for more focus on factors for increased early antenatal care utilization. Women with higher education and those with no cost problems were more likely to have early ANC utilization, skilled delivery and EPNC; therefore there is need to design and implement policies targeting social and economically disadvantaged women.

## Background

World Health Organization (WHO) and United Nations Fund for Population Activities (UNFPA) 2019, show that a pregnant woman dies somewhere in the world every 11 seconds from conditions that could have been avoided and prevented [1]. Globally, roughly 830 women world-wide still die from pregnancy complications, childbirth, or after birth effects [2–5]. Timely care makes it possible for health workers to discover and deal with any problems as soon as possible; hence a combination of early antenatal care, skilled delivery and early postnatal care have been proposed as key strategies for improving maternal and neonatal health outcomes in resource-poor countries such as Uganda [1,6–8]. The major factors associated with delayed Maternal Health Care Service (MHCS) utilization in less developed countries are related to distance to health facilities, service costs, community health workers, mass media, incomes, age and education, inadequate emotional support from husbands and weak incentives to use public health facilities [9–12].

Antenatal care (ANC) is defined as monthly visits during the first two trimesters. The first ANC visit should be within the first trimester, subsequently fortnight visits from 28th week to 36th week of pregnancy and weekly visits after 36th week until delivery at a health facility [2,4,13]. On the other hand, postnatal care is a personalized care given to a woman within 48 hours after the delivery of the placenta and continuing for 42 days [14–17]. Continuum of care is important in prevention and or reducing pregnancy and birth complications and risks that may lead to death or serious illness of the mother and her baby [5,18].

Complications like blood pressure, defects and infections, severe bleeding and miscarriages are avoidable since they can be prevented, detected, or treated during early antenatal visits, intranatal and early postnatal care by a trained health workers [19,20]. In Africa, approximately 30–40% of direct maternal deaths are due to hemorrhage and infections mostly in the postpartum period [11,12,21] and specifically within 48 hours after delivery [15,22,23]. Moreover, the postpartum period in Africa is often marked by cultural practices that keep mothers and babies indoors. Thus, if a mother becomes ill during this period, seeking formal health care is often a challenge [24–26].

Early antenatal care is vital to maternal care and treatments which depend not only on the types of examinations performed, but also on the counseling and preventive measures administered to avoid the risk of maternal and infant morbidity and or mortality [5,13,21,27,28]. During ANC, mothers are taught about the danger signs of pregnancy complications, they receive health tests and medical examinations like blood pressure measurements, tetanus vaccinations, HIV; receive folic acid and iron supplements, and are told the merits of skilled health care delivery [21,29,30].

Early postnatal care is critical to the prevention of many complications, including: postpartum hemorrhage [31,32] vertical transmission of diseases [25,33], detection and treatment of problems and complication readiness [12]. It also allows an evaluation and the development of a personalized postnatal care plan, counseling for HIV and testing, contraception and resumption of sexual activity [9,33] and referral of mother and baby for special care when necessary [5].

Although Uganda has registered slight progress in utilization of Maternal Health Care Services (MHCS), according to the Uganda Demographic Health Survey (UDHS) 2016 [5], 15 pregnant women die daily. The country still ranks among the top 40 countries in the world with high maternal mortality at 440 deaths per 100,000 live births [1,6] in comparison to the global target of 70 deaths per 100,000 live births [34]. Progress toward United Nations (UN) proposed Sustainable Development Goal 3.1, aimed at reducing maternal mortality by 2030, can be achieved by better interventions targeting early MHCS utilization [2,35].

In response, the Uganda National Population Policy and Safe Motherhood program (SMP) sought to improve maternal health by promoting informed choice [36], strategic partnerships between communities and strengthening referral systems [5,27,37–39]. Furthermore, WHO and Ministry of Health (MoH) earmarked the recommended time for the first ANC, increased strategies for a comprehensive and basic Emergency Obstetric Care (EMOC) services, availed skilled health workers and emphasized early postnatal care [2,40].

However, women often fail and or delay to seek medical assistance, which hampers WHO guidelines and MoH recommendations [5], skilled deliveries [20] and postnatal care within 48 hours [15,41]. Moreover, delayed maternal health care services utilization contribute to major complications that account for 80% of all maternal deaths [3,7,13,27,42], infections, obstructed labor, hypertensive disorders and complications of unsafe abortion [43–45]. The 2016 UDHS results showed that most expectant mothers attend their first ANC after 22 weeks of pregnancy while 26% of mothers do not deliver from a health facility and 46% of mothers do not receive postnatal care within 48 hours.

Various studies have shown that women who utilize early ANC develop confidence in the maternity system and are more likely to deliver from the health facility and obtain EPNC [21,46,47]. Therefore, the main purpose of this study is to establish the predictors associated with the interrelationships between early antenatal, health facility delivery and early postnatal care utilization in Uganda. This study contributes toward better understanding of both direct and indirect factors that will enable policy makers and women make better informed decisions.

## Methods

### Data source

The 2016 Uganda Demographic and Health Survey (UDHS) data were utilized for this study. As part of the requirements, authorization and approval to use the data were obtained from MEASURE DHS Program and ICF Macro International U.S.A, respectively. These data are from a nationally representative sample of households obtained at two-stage cluster sampling. The first stage involves the selection of cluster sample followed by selection of households. Women aged 15–49 were asked questions about their demographic and household characteristics, maternal and child health indicators. Consent was sought from respondents before data collection.

### Sample size and selection of study participants

The sample of interest for this study was 10,152 women aged 15–49 who had a live birth within 5 years preceding the survey. However, only 5,901 women who had a live birth in 2 years prior to the survey were required to respond to a question about timing of their first postnatal care.

### Endogenous variables

The three interrelated study outcome (endogenous) variables were: early antenatal care measured as timing of first antenatal visit, health facility delivery denoting supervised delivery at a health facility, and last early postnatal care measured as timing of first postnatal checkup.

### Exogenous variables

**Figure A1.**
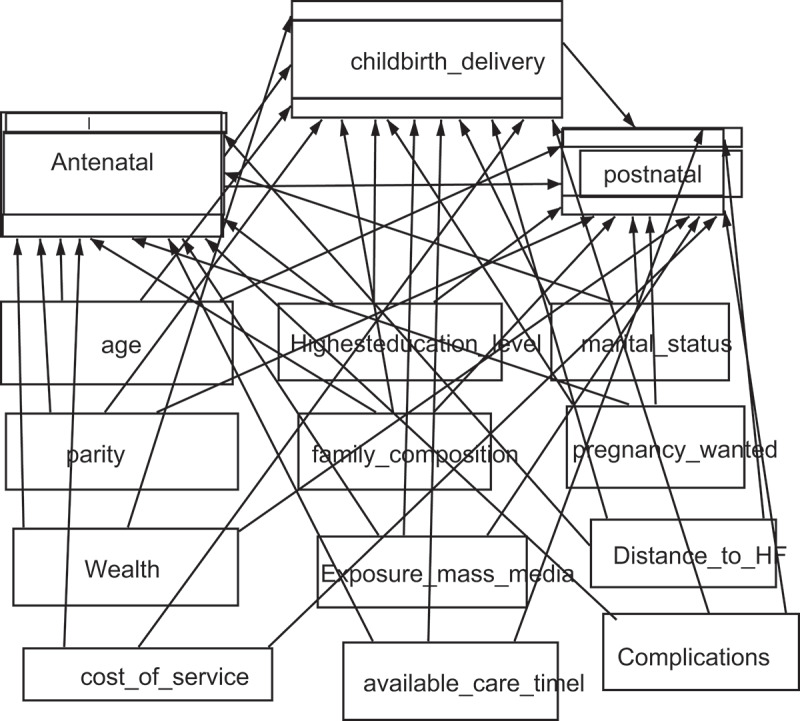
Shows the interrelationships between the three endogenous variables in the model

In this study, antenatal care, health facility delivery and postnatal care were considered as health-seeking behaviors; therefore, Andersen’s behavioral model of health service use was modified to select the predictors as shown in the conceptual framework, Figure A1. In accordance to Andersen’s model, maternal health care utilization is a function of three factors: predisposing, enabling and need factors. The predisposing factors (socio-demographic factors) in the model included marital status, parity, maternal age at last birth, maternal highest education level, exposure to mass media, distance to health facility, readily available community health workers. Enabling factors were wealth and cost of service and need factors (functional or health related) were pregnancy complications and desire for pregnancy. Table 1 shows the measurements of the variables adopted in the study.

**Table 1.** Operationalization and measurement of variables used in the study

Code	Variable	Description	Coding if any	Data type
Y1	Early Antenatal care	Timing of the first antenatal visit	1. First ANC visit within first trimester. 2. First ANC visit after first trimester	Binary
Y2	Health facility delivery	Supervised delivery at the health facility or institutional delivery	1. Delivered from a health facility 2. Did not deliver from a health facility	Binary
Y3	Early Postnatal care	Timing of first postnatal checkup	1. First PNC within 48 hours after birth 2. First PNC after 48 hours	Binary
X1	Age of the women	Age of the women at the time of the survey	1. 15–18 2. 19–35 3. 36–49	ordinal
X2	Parity	Number of children the woman has already had (CEB)	1. 1–3 2. 4–6 3. 7+	ordinal
X3	Highest maternal education level	Mother’s highest level of education	1. some primary 2. completed primary seven 3. some secondary 4. completed secondary six	ordinal
X4	Wealth	Income level of the household	1. Poor 2. middle 3. Rich	ordinal
X5	Marital status	Marital status of the woman	1. Unmarried 2. Married	nominal
X6	Pregnancy wanted	If the mother wanted the last pregnancy	1. Yes 2. No	nominal
X7	Exposure to mass media	Women who listen to radio, read newspapers or watch television	1. Exposure 2. Non exposure	nominal
X8	Pregnancy complications	If the pregnancy had complications or not	1. Yes 2. No	nominal
X9	Community factor	Availability of community health worker	1. Yes 2. No	nominal
X10	Distance to the health facility	If the distance from home to the health facility is a big problem or not.	1. Big problem 2. not big problem	nominal
X11	Direct costs/fees	If cost paid while accessing a service is a problem or not	1. big problem 2. not	nominal

### Data analysis

Data were analysed using STATA 13.0 [48]. The distribution of exogenous and endogenous variables is presented in Tables 2 and 3, respectively. Differentials in early ANC, health facility delivery, and EPNC with their corresponding predictors was undertaken using a binary logistic regression model. The purpose of this stage was to determine the significant variables which would be used in the final analysis procedure. In other words, variables that had a relatively small probability value of 0.1 or less were considered for inclusion in the generalized structural equation model (GSEM) to ascertain direct and indirect effects between the endogenous variables.

**Table 2.** Weighted percentage distribution of women by background characteristics

Characteristic	Category	Number (N = 10,152)	Percent (%)
Age	15–19	2347	23.1
	20–34	5154	50.8
	35–49	2651	26.1
Highest Maternal Education level	Some primary	4406	43.4
	Completed primary seven	1827	18
	Some secondary	3198	31.5
	Completed secondary six	721	7.1
Marital status	Married	3189	31.4
	Unmarried	6963	68.6
Wealth	Poor	4128	40.7
	Middle	1912	18.8
	Rich	4112	40.5
Parity	1–3 4–6 7+	2231 3664 4257	22 36.1 41.9
Distance to health facility	Big problem	3957	38.9
	Not big problem	6195	60.1
Cost of service	Big problem	4763	47
	Not big problem	5389	53
Community health worker	Readily available	7258	71.5
	Not readily available	2894	28.5
Media exposure	Exposed	8110	80
	Not exposed	2042	20
Pregnancy wanted	Yes	6185	60.9
	No	3967	39.1
Pregnancy complications	Yes	660	6.5
	No	9492	93.5

**Table 3.** Weighted percentage distribution of women by endogenous variables

Factor	Category	Number	Percent (%)
Early Antenatal care n = 10,152	Within first trimester	2897	28.5
	After first trimester	7255	71.5
Health facility delivery n = 10,152	Delivered from the health facility	7512	74
	Did not deliver from the health facility	2640	26
Early postnatal care n = 5,901	Within 48 hours after childbirth	3186	54
	After 48 hours after childbirth	2715	46

The net impact of the exogenous variables on each of the endogenous variables was estimated using the GSEM method. All the endogenous variables being binary; thus, the study used the GSEM logistic link and binomial family option. Missing data were treated using equation-wise deletion and sampling weights were used before analysis because of non-proportional allocation of the sample to different regions and areas of residence. Sampling weights ensured the representativeness of the results at both the national and regional levels.

The structural equation system is as follows: Equations (1), (2) and (3) describe the relationship between early ANC utilization and associated predictors, health facility delivery and associated predictors as well as EPNC and associated predictors respectively.

(1) $$\ln\left[\frac{pr\left(Y_{1}=1\right)}{1-pr\left(Y_{1}=1\right)}\right]=\;\beta_{0}+{\tilde{\beta }}_{i}\;{\tilde{X}}_{i}+u_{i}$$

(2) $$\ln\left[\frac{pr\left(Y_{2}=1\right)}{1-pr\left(Y_{2}=1\right)}\right]=\beta_{0}+\beta_{1}Y_{1}+{\tilde{\beta }}_{j}\;{\tilde{X}}_{j}+u_{j}$$

(3) $$\ln\left[\frac{pr\left(Y_{3}=1\right)}{1-pr\left(Y_{3}=1\right)}\right]=\beta_{0}+\beta_{1}Y_{1}+\beta_{2}Y_{2}+{\tilde{\beta }}_{k}\;{\tilde{X}}_{k}+u_{k}$$

Where; $Y_{i}\;for\;i=1,2,3$ were the endogenous variables, $\beta_{0}$ were intercepts of the models, $\overset{i}{\beta },\overset{j}{\beta },\overset{k}{\beta }$ the matrices of the slope coefficients, $\overset{i}{X},\overset{j}{X},\overset{k}{X}$ the matrices of independent variables, and $u_{i},u_{j},u_{k}$ the error terms.

According to Bollen and Long [49], structural refers to the assumption that the parameters are descriptive measures of association and reveal an invariant causal relation. Furthermore, both direct and indirect effects are estimated. An indirect effect occurs when an exogenous variable affects an endogenous variable through another endogenous variable [50].

Regression diagnostics tests of multicollinearity and goodness-of-fit of the model were established. Multicollinearity was tested by generating the uncentered Variance Inflation Factors (VIF) after fitting a logistic regression for each endogenous variable and Akaike Information Criterion (AIC) test was used for testing the goodness-of-fit.

## Results

This section examines descriptive characteristics of women, the binary logistic regression and GSEM models procedures. First, results of the weighted percentage distribution of women by background characteristics and endogenous variables are presented. Second, a binary logistic regression model was fitted to examine the unadjusted odds ratio of each category of the exogenous variable on all indicators. This was done to ascertain the variables that had a relatively small probability value of 0.1 or less to be considered for inclusion in the GSEM. Lastly, the generalized structural equation model was run on all variables with exception of the reference categories.

### Descriptive characteristics of women

Table 2 presents the characteristics of respondents, who are summarized as follows: Few women (26.1%) were of aged more than 35 years; very few women completed secondary six (7.1%) education. More than half of the women were unmarried (68.6%); poor and rich women were almost equal (40.7% and 40.5%, respectively). About six-in-every ten women (61%) didn’t point out distance to health facility as a big problem. There was a slight difference between women with cost of service problems and those without (47% versus 53%, respectively); most health workers were readily available in the community (71.5%). More than three quarters of women were exposed to at-least one form of media (80%). More than half of the women wanted the pregnancy (61%) and very few had pregnancy complications (6.5%). Finally, most women reported an average of 3 children ever born with a standard deviation of 3 children.

Results in Table 3 reveal that few women reported receiving first ANC (28.5%) within the first trimester and more than half of women delivered from a health facility (74%). About 56% of women indicated receiving postnatal checkup within 48 hours after childbirth (54%).

## Bivariate and multivariate results

Table 4 presents results of predictors associated with early ANC utilization as a mediating factor for health facility delivery and EPNC. Tables 5 and 6 present the direct predictors associated with health facility delivery and EPNC.

**Table 4.** Logit and GSEM models predicting the Odds Ratios of women utilizing early ANC

Characteristic	Category	unadjusted OR (95% CI)	Adjusted OR (95% CI)
Age	15–19 (r)	0.89 (0.81–0.94)*	
	20–34	1.31 (1.12–1.42)*	0.92 (0.79–0.97)**
	35–49	0.97 (0.88–1.05)	1.18 (1.10–1.35)**
Highest Maternal Education level	Some primary (r)	1.32 (0.92–1.40)	
	Completed primary seven	1.69 (1.45–1.84)*	1.68 (1.56–1.84)**
	Some secondary	0.70 (0.64–0.96)*	0.90 (0.80–1.10)
	Completed secondary six	1.13 (0.93–1.05)	1.17 (0.94–1.35)
Marital status	Unmarried (r)	0.85 (0.75–0.94)*	
	Married	1.24 (1.19–1.43)*	1.12 (0.94–1.26)**
Wealth	Poor (r)	0.90 (0.87–1.1)	-
	Middle	0.86 (0.71–1.02)	-
	Rich	0.88 (0.78–1.05)	-
Distance to health facility	Big problem (r)	0.87 (0.89–0.95)*	
	Not big problem	1.51 (1.23–1.65)*	1.35 (1.23–1.73)**
Cost of service	Big problem (r)	0.61 (0.47–0.82)*	
	Not big problem	1.13 (1.10–1.32)*	1.85 (1.31–2.12)**
Community health worker	Not readily available (r)	1.05 (1.03–1.19)*	
	Readily available	1.13 (1.08–1.35)*	1.06 (1.04–1.17)**
Media exposure	Not exposed (r)	0.9 (0.75–1.13)	-
	Exposed	1.08 (0.98–1.31)	-
Pregnancy wanted	No (r)	0.82 (0.76–0.98)*	
	Yes	1.17 (1.05–1.38)*	1.15 (1.07–1.36)**
Pregnancy complications	No (r)	0.65 (0.45–0.87)*	
	Yes	1.95 (1.73–2.45)*	2.04 (1.85–2.26)**
Parity	1–3(r)	0.76 (0.65–0.86)*	0.71 (0.62–0.80)**
	4–6	0.86 (0.78–1.1)	0.81 (0.72–1.00)
	7+	1.2 (0.92–1.40)	1.03 (0.96–1.23)

(OR): odds ratios; CI: confidence intervals; * indicates variables with p < 0.1, ** indicates significant effects with p < 0.05, and (r) indicates the reference category and (-) indicates variables not considered at multivariable level

**Table 5.** Logit and GSEM model predicting the Odds Ratios of women utilizing a health facility

Characteristic	Category	unadjusted OR (95% CI)	Adjusted OR (95% CI)
Early ANC	Went after the first trimester	1.10 (1.05–1.17)*	
	Went within first trimester	1.08 (1.03–1.15)*	1.04 (1.01–1.14)**
Age	15–19 (r)	1.23 (1.10–1.40)*	
	20–34	0.87 (0.78–0.94)*	0.83 (0.79–0.97)**
	35–49	1.57 (1.32–2.10)*	1.52 (1.32–2.05)**
Highest Maternal Education level	Some primary (r)	1.52 (1.31–1.76)*	
	Completed primary seven	2.47 (2.29–2.68)*	1.47 (1.29–1.68)**
	Some secondary	2.20 (1.87–2.58)*	2.20 (1.87–2.58)**
	Completed secondary six	3.45 (2.64–4.45)*	3.58 (2.68–4.44)**
Marital status	Unmarried (r)	1.17 (0.95–1.32)	
	Married	1.12 (0.92–1.26)	-
Wealth	Poor (r)	1.01 (0.89–1.50)	
	Middle	1.12 (0.99–1.26)	1.21 (0.89–1.34)
	Rich	1.67 (1.50–1.84)*	1.15 (0.78–1.29)
Distance to health facility	Big problem (r)	0.81 (0.75–1.01)	
	Not big problem	1.23 (1.00–1.34)	-
Cost of service	Big problem (r)	0.96 (0.81–1.07)	
	Not big problem	0.87 (0.88–1.12)	-
Community health worker	Not readily available (r)	1.08 (1.04–1.24)*	
	Readily available	2.01 (1.94–2.56)*	2.05 (1.87–2.47)**
Media exposure	Not exposed (r)	0.90 (0.81–0.97)*	
	Exposed	1.06 (0.97–1.31)	1.26 (1.14–1.40)**
Pregnancy wanted	No (r)	0.85 (0.79–1.12)	
	Yes	0.95 (0.89–1.08)	-
pregnancy complications	No (r)	0.82 (0.72–1.06)	
	Yes	1.27 (0.97–1.47)	-
Parity	1–3(r) 4-6 7+	1.10 (0.93–1.24) 0.89 (0.80–105) 0.94 (0.882–0.97)*	0.95 (0.82–1.07) 0.71 (0.67–1.02) 0.92 (0.86–0.95)**

(OR): odds ratios; CI: confidence intervals; * indicates variables with p < 0.1, ** indicates significant effects with p < 0.05, and (r) indicates the reference category and (-) indicates variables not considered at multivariable level

**Table 6.** Logit and GSEM model predicting the Odds Ratios of women utilizing early PNC

Characteristic	Category	unadjusted OR (95% CI)	Adjusted OR (95% CI)
Health facility delivery	Did not deliver from health facility Delivered from health facility	0.87 (0.85–1.08) 1.50 (1.41–1.87)*	0.98 (0.87–1.13)
Early ANC	Went after first trimester	0.80 (0.67–0.92)*	
	Went within the first trimester	1.12 (1.07–1.23)*	1.10 (1.05–1.26)*
Age	15–19 (r)	1.89 (1.76–1.98)*	
	20–34	0.97 (0.88–0.14)	0.91 (0.84–1.21)
	35–49	1.27 (1.17–1.40)	1.14 (0.94–1.35)
Highest Maternal Education level	Some primary (r)	0.89 (0.73–1.23)	
	Completed primary seven	0.80 (0.68–1.18)	1.04 (0.92–1.27)
	Some secondary	1.13 (0.92–1.38)*	1.19(1.04–1.38)*
	Completed secondary six	1.42 (1.00–1.82)	1.11 (0.94–1.35)
Marital status	Unmarried (r)	1.23 (0.98–1.51)	-
	Married	1.02 (0.87–1.16)	-
Wealth	Poor (r)	0.69 (0.59–0.81)*	
	Middle	0.81 (0.72–1.16)	0.74 (0.68–1.34)
	Rich	0.93 (0.98–1.34)	1.24 (1.13–1.34)*
Distance to health facility	Big problem (r)	0.91 (0.72–0.99)*	
	Not big problem	1.34 (1.17–1.67)*	1.90 (1.53–2.35)*
Cost of service	Big problem (r)	0.87 (0.76–0.96)*	
	Not big problem	0.98 (0.88–1.12)	0.92 (0.85–1.16)
Community health worker	Not readily available (r)	0.85 (0.75–0.94)*	
	Readily available	2.06 (1.88–2.45)*	1.15 (1.03–1.25)*
Media exposure	Not exposed (r)	0.87 (0.78–1.42)	
	Exposed	1.51 (1.24–1.85)*	0.83(0.72–1.21)
Pregnancy wanted	No (r)	0.89 (0.80–1.10)	
	Yes	1.03(0.93–1.28)	-
pregnancy complications	No (r)	1.14 (0.91–1.33)	
	Yes	1.18 (1.07–1.67)*	1.17 (1.05–1.31)*
Parity	1–3 4-6 7+	0.76 (0.61–1.01) 0.92 (0.84–1.2) 0.82 (0.73–0.93)*	0.70 (0.61–1.1) 0.86 (0.78–1.09) 0.73 (0.67–0.81)**

(OR): odds ratios; CI: confidence intervals; * indicates variables with p < 0.1, ** indicates significant effects with p < 0.05, and (r) indicates the reference category and (-) indicates variables not considered at multivariable level

The bivariate relationships between each exogenous variables and early ANC utilization show that, all variables were significant (p < 0.1) with the exception of wealth and media exposure; hence, included in the multivariable analysis. Results of the GSEM model as indicated in Table 4 show that, holding other factors constant, increased odds of early ANC utilization were associated with: adult women aged 34–49 years compared to teenage women, having completed primary seven compared to some primary, being married, having no distance to a health facility and cost of service issues, having available community health workers, desire for pregnancy and having a complicated pregnancy. However, women aged 20–34 and low parity had reduced odds of early ANC utilization.

The bivariate relationships between each exogenous category and health facility delivery show that, early ANC, age, highest maternal education level, wealth, community health workers, media exposure and parity were significant factors (p < 0.1); hence, included in the multivariable analysis.

Results of the GSEM in Table 5 further revealed that, women who accessed ANC within the first trimester had a 4% increased odds of deliver from a health facility compared to women who had delayed first ANC (OR = 1.04; 95% CI = 1.01–1.14). Women aged 20–34 years had 17% reduced odds, and adult women aged 35–49 had 52% increased odds (OR = 0.83; 95% CI = 0.79–0.97 & 1.52; 95% CI = 1.32–2.05, respectively) of delivery from a health facility when compared to teenage women. Completing primary 7 (OR = 1.47; 95% CI = 1.29–1.68), secondary education (OR = 2.2; 95% CI = 1.87–2.58) and Senior Six (OR = 3.6; 95% CI = 2.68–4.44) education was associated with increased the odds of delivery from a health facility when compared to women having some Primary education. Women in communities with readily available health workers were twice as likely to deliver from a health facility compared to women in communities without readily available health workers (OR = 2.05; 95% CI = 1.87–2.47). Women exposed to at-least one form of media had 26% increased odds of delivery from a health facility when compared to women not exposed (OR = 1.26; 95% CI = 1.14–1.40). Lastly, a higher number of children ever born was associated with reduced odds of delivery from a health facility on average by 8% (OR = 0.92; 95% CI = 0.86–0.95).

The bivariate relationships between each exogenous category and early PNC show that all variables were significant (p < 0.1) with the exception of marital status and pregnancy wanted; hence, included from the analysis in the multivariable analysis.

Results of the GSEM model as indicated in Table 6 show that, holding other factors constant, women having some secondary education level had 19% increased odds to utilize EPNC as compared to women with having some primary (OR = 1.19; 95% CI = 1.04–1.38). Rich women had a 24% increased odds EPNC utilization as compared to poor women (OR = 1.24; 95% CI = 1.13–1.34). Women with no distance to a health facility issues had 90% increased odds of EPNC utilization when compared to women with distance issues (OR = 1.9; 95% CI = 1.53–2.35). Women in communities with readily available health workers had 15% increased odds of EPNC utilization compared to women in communities without readily available health workers (OR = 1.15; 95% CI = 1.03–1.25). Women with complications had 17% increased odds of EPNC utilization compared to women who had no pregnancy complications (OR = 1.17; 95% CI = 1.05–1.31). Last, a higher number of children ever born lowered the odds EPNC utilization on average by 7% (OR = 0.73; 95% CI = 0.67–0.81).

### Predictors on health facility delivery and early postnatal care utilization through early ANC

Notably, an endogenous variable early ANC, had a significant impact on health facility delivery and EPNC utilization (OR = 1.04 & 1.10, p < 0.05 respectively). This implies that insignificant predictors of health facility delivery and EPNC utilization that are significantly associated with early ANC utilization indirectly impact health facility delivery and EPNC utilization. Table 7 presents the indirect effects on health facility delivery and EPNC through early ANC utilization.

**Table 7.** Indirect effects on health facility delivery and EPNC through early ANC

Characteristic	Category	health facility delivery	Early PNC
Age	15–19		
	20–34		(1.01*1.1) = 1.01*
	35–49		
Highest Maternal Education level	Some primary		
	Completed primary seven		(1.69*1.1) = 1.85*
	Some secondary		
	Completed secondary six		
Marital status	Unmarried		
	Married	(1.04*1.1) = 1.16*	
Distance to health facility	Big problem		
	Not big problem	(1.04*1.35) = 1.40*	
Cost of service	Big problem		
	Not big problem		(1.85*1.1) = 2.04*
pregnancy complications	No (r)		
	Yes	(1.04*2.04) = 2.12*	

* indicates significant effects with p < 0.05

Results presented in Table 7 revealed that married women who utilized first ANC within first trimester had 16% increased odds of delivery from a health facility compared to married women who utilized first ANC after first trimester. Women with no distance problems and who utilized first ANC within first trimester had 40% increased odds of delivery from a health facility compared to women with no distance issues but accessed first ANC after the first trimester. Women who utilized ANC early with complicated pregnancy were twice likely to deliver from a health facility compared to women who had complications and delayed first ANC visit.

In addition, the odds to deliver from a health facility for adult women aged of 35–49 who utilized early ANC were not significantly different from adult women aged 35–49 who delayed first ANC visit. Women who had early ANC without cost of service issues were twice as likely to utilize EPNC compared to women with no cost of service problems but delayed first ANC visit. The odds of utilizing EPNC among women having complete primary seven and utilized early ANC increased significantly by 85% compared to those who utilized ANC after the first trimester.

### The GSEM model

**Figure 1.**
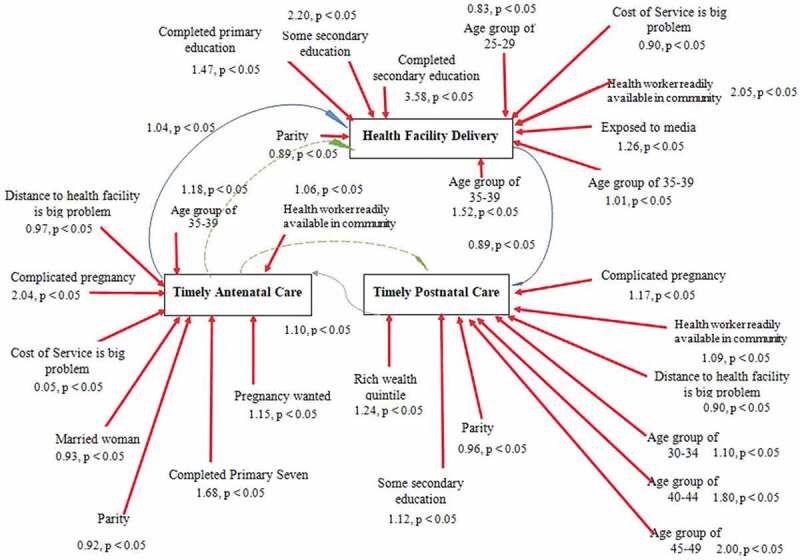
Causal loop showing predictors of early ANC, health facility delivery and EPNC

Figure 1 summarizes the structural equation analysis on the relationship between early ANC, health facility delivery and EPNC.

## Discussion

The study found out that interrelationships between early ANC, health facility delivery and EPNC exist. Primarily, since early ANC had a statistically significant effect on health facility delivery and EPNC, indirect factors are evident among predictors of health facility delivery and early postnatal care.

Though majority of Ugandan women receive antenatal care from a skilled provider [5], results from this study revealed that very few women access their first antenatal care visit within the first trimester. Significant delays for the first ANC visit have been observed in other countries including Rwanda [51] and Ethiopia where more than half of women had delayed ANC in 2012 [52]. Results further reveal that women who are likely to delay first antenatal care visit are teenage women, those who have low education level, women in communities with no health workers, women with no complications, and those with distance to a health facility and costs of services issues. In line with this, women often delay first antenatal care [2,27,44,53]; moreover first trimester is a vital period when medical personnel check the mother’s medical history, like if a mother had a history of an ectopic pregnancy to avoid reoccurrence, test for HIV, check for infections and or defects and blood pressure [20,33,54]. Women require a better understanding on how to improve and benefit from early medical checkup during pregnancy, at birth and after childbirth for better neonatal and mother outcomes.

### Interrelationship between early ANC, health facility delivery and EPNC

The direct determinants for increased odds of early ANC utilization as a mediating factor for health facility delivery and EPNC were: adult women aged 35–49, having completed primary seven, distance to a health facility and costs of services not being a problem, available community workers, pregnancy complications and desire for pregnancy. Women aged 20–34 and parity reduced odds of early ANC utilization.

The study agrees with [13,53,55–57] where family income, age at delivery, media exposure, attitude toward pregnancy, knowledge about the danger signs of pregnancy, husband’s approval of ANC and distance to health facility were associated with ANC service utilization at any point during pregnancy. Hagey et al. [58], explored social and behavioral factors that affect early initiation of ANC from the perspective of health care providers in Kigali city and found that women’s knowledge gaps and having previous births were among the main barriers to ANC initiation.

Although most studies identify direct factors influencing utilization of a health facility and early postnatal care [11,15,27,47,58–63], findings from this study further established predictors that indirectly affect utilization of a health facility and EPNC utilization. Through early ANC, being married, no distance issues and complications indirectly influenced delivery at a health facility. In addition, women aged 20–34, completing primary seven and no cost problems indirectly influenced EPNC through early ANC. A study by Machira and Palamuleni [21] revealed that women who obtained early ANC in Malawi were more likely to utilize public health care childbirth and EPNC. Grigg et al. [64], revealed that confidence develops in women to deliver from a health facility given that they had attained ANC. This is because they become confident with the midwife, the maternity system as well as the birth process. Furthermore, El Shiekh and van der Kwaak [47] reported that nomads in Sudan who utilized health facility had had early and at-least 4 ANC visits compared to their counterparts. However, Pell et al. [42] disagreed that early antenatal attendance may discourage delivery in health units if mothers are told that the pregnancy is normal.

Married women who utilized first ANC within first trimester had increased odds of deliver from a health facility as compared to married women who utilized first ANC after first trimester. Women with no distance problems who utilized first ANC within first trimester had increased odds of delivery from a health facility as compared to women with no distance issues but accessed first ANC after the first trimester. Women who utilized early ANC with complicated pregnancy were more likely to deliver from a health facility compared to women who had complications and delayed first ANC visit.

In addition, women who utilized early ANC without cost of service problems were twice more likely to utilize EPNC compared to women with no cost of service problems but delayed first ANC visit. The odds of utilizing EPNC among women having completed primary seven and utilized early ANC were significantly higher when compared to women who utilized ANC after the first trimester. The odds of delivery from a health facility for adult women aged of 35–49 who utilized early ANC were not significantly different from those who delayed their first ANC visit.

The results generally indicate that there are significant interrelationships between maternal healthcare services in Uganda, as shown by both direct and indirect effects. Notably, early ANC is vital in determining utilization of a health facility and EPNC. It is however puzzling that, at a time Ugandan women seem to be doing well in terms of delivery at a health facility, EPNC checkup is still low. This implies that there are women who deliver from a health facility, but never undertake EPNC to be a very critical service to deal with post-birth complications.

In sum, the GSEM results reveal the interconnectedness between early ANC, health facility delivery, EPNC, and their predictors (age of mother, maternal education level, wealth, marital status, costs of service, distance to the health facility, availability of health worker in the community, complications and parity). This suggests that more than direct factors are accountable for the differences in the decisions of utilization of MHCS by women in Uganda. Indeed, this knowledge is a requisite material for policy options in addressing the challenges Uganda faces in its efforts to ensure improvement of maternal health through improved timely utilization of maternal health services.

### Limitation of the study

The major limitation of this study relates to the secondary nature of the data that were used. Invariably many events captured through a retrospective inquiry are often susceptible to recall bias and memory lapse. For example, information asked concerning time for first antenatal care visit might not be remembered exactly, which could impact on precision of the study findings. Furthermore, only reports of women who were alive at the time of the survey were obtained. Moreover, in populations where maternal mortality ratios are very high, women of high mortality risk could have succumbed to the force of mortality.

## Conclusion

Early antenatal care was a mediating factor for health facility delivery and early postnatal care; hence, in order to improve maternal and neonatal health outcomes, there is need to formulate policies and design maternal health service programs that integrate early antenatal, health facility delivery and early postnatal care utilization.

Social and economically disadvantage women should be financially supported by reducing the costs of attaining health services. Government of Uganda should enact the bill on insurance, encourage pregnancy centering and strengthen the position of community health workers.

Female child education completion, scholarship programs and legislation against early marriages should further be enforced in Uganda, so that young women can remain in school longer. Educated women are better positioned to acquire, understand and utilize knowledge when exposed to media on maternal health information [65–69].

## Data Availability

The datasets generated and/or analyzed during this study are not publicly available due to a requirement of approval from ICF Macro International U.S.A to use the data. The authors were authorized to use ‘Survey’ data from the Demographic and Health Surveys (DHS) Program accessed on: http://www.dhsprogram.com/data/dataset_admin/login_main.cfm.

## Paper context

Research overwhelmingly shows that mothers seeking health services during pregnancy, at birth and after birth have better maternal and neonatal outcomes [13, 23, 27, 66-70], however, most scholars address Maternal Healthcare Service (MHS) indicators independently. To tackle this problem, we explored the interrelationships between early antenatal care, health facility delivery and early postnatal care, thus a giving broader understanding of both direct and indirect predictors of selected MHS for better women awareness and policy formulation.

## Appendix

**APPROVAL LETTER**

You have been authorized to download ‘Survey’ data from the Demographic and Health Surveys (DHS) Program. To begin downloading, please login at: http://www.dhsprogram.com/data/dataset_admin/login_main.cfm. If you are new to DHS Datasets, and need additional guidance, please watch our videos on:

Downloading Datasets – https://youtu.be/Kzv075WRVZA

Bulk Dataset Download – https://youtu.be/bVfQ_4ZxBAQ

The requested data should only be used by you, and for the purpose of the registered research or study. The data must not be passed on to others, without the written consent of DHS. To use the data for another purpose, a new research project must be ‘created’ in your account. All DHS data should be treated as confidential, and no effort should be made to identify any household or individual respondent interviewed in the survey. Users are required to submit a copy of any reports/publications resulting from using the DHS data files to:archive@dhsprogram.com. Please reference the complete terms of use at: https://dhsprogram.com/Data/terms-of-use.cfm.

The files you will download are in zipped format and must be unzipped before analysis. After unzipping, please print the file with the.DOC/DOCX extension (found in the Individual and Male Recode Zips). This file contains useful information on country specific variables and differences in the Standard Recode definition. You will also need the DHS Recode Manual:http://dhsprogram.com/publications/publication-dhsg4-dhs-questionnaires-and-manuals.cfm. This manual contains a general description of the recode data file, including the rationale for recoding; a description of coding standards and recode variables, and a listing of the standard dictionary, with basic information relating to each variable.

It is essential that you consult the questionnaire for the country, when using the data files. Questionnaires are in the appendices of each survey’s final report: http://dhsprogram.com/publications/publications-by-type.cfm. We also recommend that you make use of the Data Tools and Manuals at: http://www.dhsprogram.com/accesssurveys/technical_assistance.cfm.

For problems with your user account, please email archive@dhsprogram.com. For data related questions, please register to participate in the DHS Program User Forum at:http://userforum.dhsprogram.com.

The Demographic and Health Surveys (DHS) Program

ICF

530 Gaither Road

Suite 500

Rockville, MD 20850

USA

LOGIN INFORMATION:

Login Email: ratuhaire@mubs.ac.ug

Password: (use password selected when you registered)
